# Multiple Sources of Surprisal Affect Illusory Vowel Epenthesis

**DOI:** 10.3389/fpsyg.2021.677571

**Published:** 2021-08-31

**Authors:** James Whang

**Affiliations:** Department of Linguistics, Seoul National University, Seoul, South Korea

**Keywords:** illusory vowel epenthesis, information theory, Japanese, phonology, phonotactic learning, alternation learning

## Abstract

Illusory epenthesis is a phenomenon in which listeners report hearing a vowel between a phonotactically illegal consonant cluster, even in the complete absence of vocalic cues. The present study uses Japanese as a test case and investigates the respective roles of three mechanisms that have been claimed to drive the choice of epenthetic vowel—phonetic minimality, phonotactic predictability, and phonological alternations—and propose that they share the same rational goal of searching for the vowel that minimally alters the original speech signal. Additionally, crucial assumptions regarding phonological knowledge held by previous studies are tested in a series of corpus analyses using the Corpus of Spontaneous Japanese. Results show that all three mechanisms can only partially account for epenthesis patterns observed in language users, and the study concludes by discussing possible ways in which the mechanisms might be integrated.

## 1. Introduction

Illusory epenthesis, or perceptual epenthesis, is a phenomenon where listeners perceive C_1_C_2_ consonant clusters that are phonotactically illegal in their native language as C_1_VC_2_ sequences (Dupoux et al., 1999, 2011; Dehaene-Lambertz et al., 2000; Monahan et al., 2009; Durvasula and Kahng, 2015; Whang, 2019; Kilpatrick et al., 2020). The misperceived medial vowel is not present in the original speech signal but makes the resulting sequence phonotactically legal. Since C_1_C_2_ sequences are repaired to C_1_VC_2_ during perception, listeners have difficulties distinguishing such vowel-less vs. vowel-ful pairs accurately. For example, a series of studies by Dupoux et al. (1999, 2011) showed that Japanese listeners are unable to distinguish pairs such as [ebzo] and [ebuzo] reliably and exhibit a strong tendency toward perceiving both as [ebuzo]. Mainly three separate mechanisms have been proposed in the literature as driving the epenthetic process—phonetic minimality, phonotactic predictability, and phonological alternations. The current study investigates each in detail and shows that separately the mechanisms can only partially predict human epenthetic behavior and need to be integrated. In order to integrate the three mechanisms, the current study takes a rational approach, reframing illusory epenthesis as an optimization process (Anderson, 1990).

Rational analysis uses probabilistic approaches (e.g., Bayesian, information theoretic, and game theoretic frameworks) to explain the mechanisms that underlie human cognition. In linguistic research, the rational framework has been applied at various linguistic levels, such as pragmatic reasoning (Frank and Goodman, 2012; Lassiter and Goodman, 2013), word recognition (Norris, 2006), and speech perception (Feldman and Griffiths, 2007; Sonderegger and Yu, 2010). Of particular relevance to the current study are previous works that take an information theoretic approach to phonological processing, showing that speakers perform various manipulations at the loci of sudden surprisal peaks in the speech signal, making syllables longer or more prosodically prominent presumably to make processing easier for the listener (Aylett and Turk, 2004; Hume and Mailhot, 2013). Illusory epenthesis occurs when phonotactic violations are detected (i.e., between high surprisal sequences). The process, therefore, is simply another strategy for smoothing extreme surprisal peaks under a rational approach, and the three epenthesis mechanisms constitute different linguistic levels that a listener relies on to select the most probable output for a given input.

### 1.1. Phonetic Minimality

Phonetic minimality is the idea that the vowel that is physically shortest in a given language, and thus acoustically the closest to zero (∅; lack of segment), is used as the default epenthetic segment (Steriade, 2001; Dupoux et al., 2011). In the case of Japanese, this vowel is [u], which has an average duration of ~50 ms but can be as short as 20 ms (Beckman, 1982; Han, 1994; Shaw and Kawahara, 2019). Dupoux et al. (2011) also found that in Brazilian Portuguese, where [i] is the shortest vowel, it is [i] that functions as the default epenthetic segment in the language instead of [u] as in Japanese, further bolstering the idea that phonetically minimal vowels are the default epenthetic segment in perceptual repair.

When framed rationally, the phonetic minimality account is arguing that listeners are selecting the most probable output based on acoustic similarity. As can be seen in (1), where *A* denotes the acoustic characteristics of the input [ebzo], the output that is most consistent with the input is naturally the faithful one, namely [ebzo]. However, [ebzo] is phonotactically illegal in Japanese. It has a near-zero probability in the language and is eliminated, denoted with a strikeout. The Japanese listener, therefore, assigns the highest probability to [ebuzo] instead because [u] is the shortest vowel in Japanese, and its epenthesis results in an output that conforms to the phonotactics of the language with the smallest possible acoustic change from the original signal.

(1) Given [ebzo]:P(ebzo|A)>P(ebuzo|A)>P(ebizo|A)

The main weakness of the phonetic minimality account is that it incorrectly predicts the use of only one epenthetic segment in a given language, contrary to the fact that languages often employ more than one epenthetic vowel for phonotactic repair (e.g., Japanese—Mattingley et al., 2015; Korean—Durvasula and Kahng, 2015; Mandarin—Durvasula et al., 2018).

### 1.2. Phonotactic Predictability

Phonotactic predictability is the idea that the most frequent, and thus the most predictable, vowel in a given phonotactic context is the vowel that is epenthesized for perceptual repair. A recent study by Whang (2019) showed that while Japanese listeners do repair consonant clusters primarily through [u] epenthesis, there is also a consistent effect of the palatal consonants [ʃ, ç], after which [i] is epenthesized instead. The study calculated surprisal values (Shannon, 1948) for /u, i/ after the consonants that were used as stimuli ([b, ɡ, z, p, k, ʃ, ɸ, s, ç]) using the Corpus of Spontaneous Japanese. Crucially for the present study, Whang (2019) did not include non-high vowels and long vowels in the calculations under the assumption that Japanese listeners only consider short high vowels for epenthesis due to a lifelong experience of having to recover devoiced/deleted high vowels. The results showed that [i] had lower surprisal than [u] after the two palatal consonants [ʃ, ç] while [u] had lower surprisal after the rest. Based on these results, Whang argued that the choice between [u, i] must be driven at least in part by phonotactic predictability, that [u] is the default epenthetic segment in Japanese not only because it is the shortest vowel but also because it is the most common vowel in most contexts. When another vowel has lower surprisal in a given context (e.g., [i] after palatal consonants), the vowel with the lower surprisal is epenthesized instead, suggesting that phonotactic information can override the use of a phonetically minimal segment. Kilpatrick et al. (2020) also found similar results with [ɡ, ʧ, ʃ], where [u] was epenthesized more often after [ɡ] but [i] was epenthesized after the palatalized consonants [ʧ, ʃ].

When framed rationally, the phonotactic predictability account appeals to an idealized optimal listener's knowledge of context-specific segmental frequency to select the most probable output for a given input. This can be summarized as in (2) and (3), where relative probabilities are assigned according to the listener's knowledge of native phonotactics *K*_*p*_ rather than the physical signal *A* as was the case for phonetic minimality in (1). Since heterorganic clusters such as [bk, ʃp] are prohibited, the Japanese listener assigns near-zero probabilities to the faithful outputs, namely [ebko] and [eʃpo]. The listener then selects alternative candidates that contain the most frequent vowel in a given context. As shown in (2), [u] is the most frequent vowel after [b], whereas (3) shows that [i] is the most frequent after [ʃ]. This results in the outputs [ebuko] and [eʃipo], respectively. Note that in the case of (3), the phonetic minimality account would incorrectly predict [eʃupo] as the perceived output.

(2) $$\begin{array}{l} \text{Given }\left[ebko\right]:\\ P\left(ebuko|K_{p}\right)>P\left(ebiko|K_{p}\right)>P\left(ebko|K_{p}\right) \end{array}$$

(3) $$\begin{array}{l} \text{Given }\left[eSpo\right]:\\ P\left(eSipo|K_{p}\right)>P\left(eSupo|K_{p}\right)>P\left(eSpo|K_{p}\right) \end{array}$$

The main weakness of the phonotactic predictability account is not necessarily inherent to the approach itself but lies instead in the specific assumptions that previous studies have made. First, both Whang (2019) and Kilpatrick et al. (2020) assume *a priori* that only high vowels are considered for perceptual epenthesis due to knowledge of high vowel devoicing, and fail to show empirically that non-high vowels do not participate in illusory epenthesis. Second, and more importantly, the two studies do not distinguish voiced and devoiced vowels before calculating predictability, assuming that they belong to the same underlying vowel. In other words, voiced and devoiced vowels are assumed to be allophones of each other that alternate depending on the context. This means that as it currently stands the phonotactic predictability account subsumes phonological alternations, making it difficult to tease apart the independent effects of the two types of linguistic knowledge.

### 1.3. Phonological Alternations

Phonological alternations are different from both phonetic minimality and phonotactic predictability in that it requires a lexicon that is detailed enough to keep track of the different ways in which words and morphemes show variation on the surface. For example, an English learner must know that the suffix *-s* after nouns means “more than one” before learning that the suffix has multiple surface forms [-s, -z, -əz] depending on the final segment of the noun stem it attaches to. The phonological alternation mechanism is such that a language user learns that certain units alternate in the lexicon as a result of various phonological processes and represents the alternations as equivalent in certain contexts. An example of this sort of context-specific phonological equivalence effect on speech perception can be found in Mandarin Chinese listeners. Mandarin Chinese has four lexical tones—high (55), rising (35), contour (214), and falling (51), where the numbers in parenthesis indicate the relative pitch of the tone on a five-level scale—and has a well-known tone sandhi process where the contour tone becomes a rising tone when another contour tone immediately follows. Huang (2001) tested whether Mandarin Chinese listeners' experience with the contour-rising tone alternation in their native grammar yields different perceptual patterns from American English listeners, who have no such experience. The results showed that Mandarin Chinese listeners had more difficulties distinguish the contour and rising tones than American English listeners, suggesting that the two tones are represented as being equivalent by Mandarin Chinese listeners in certain contexts. The need for phonological alternations in explaining perceptual epenthesis was perhaps most clearly shown in a series of experiments with Korean listeners by Durvasula and Kahng (2015). Korean phonotactic structure prohibits consonant clusters within a syllable, and Korean listeners typically repair illicit clusters by epenthesizing the high central unrounded vowel [ɨ] (e.g., [klin] → [kɨlin] “clean”). However, Durvasula and Kahng (2015) found that in contexts where another vowel other than [ɨ] more frequently alternates with zero in the lexicon, it is the more frequently alternating vowel that is perceived instead. To illustrate how a vowel alternates with zero in Korean, consider the phrase “although (it is) big.” The phrase is a bimorphemic word in Korean /k^h^ɨ + ədo/ “big + although,” but /ɨə/ sequences that result from such adjectival morpheme concatenations undergo simplification. The first vowel /ɨ/ is deleted, deriving the output [k^h^ədo], resulting in a regular alternation between [ɨ] and zero after [k]. [ɨ] is actually illegal in Korean after palatal fricatives such as [ʃ], and the vowel that most frequently alternates with zero in these contexts instead is [i]. What Durvasula and Kahng (2015) found was that it is this sort of phonological alternation that best predicts the identity of the perceptually epenthesized vowel—generally [ɨ] but [i] after palatal fricatives where [ɨ] is phonotactically illegal, and either [ɨ, i] after palatal stops where both vowels are allowed—and argue that sublexical mechanisms (i.e., phonetic minimality and phonotactic predictability) are employed hierachically, becoming active only when phonological alternations fail to provide an optimal candidate for epenthesis.

The basic rational framing for phonological alternations is similar to that of phonotactic predictability, where the listener relies on a particular kind of phonological knowledge to select the optimal output for a given input. However, instead of surface level phonotactics *K*_*p*_, the listener relies on the knowledge of phonological alternations *K*_*a*_ to assign probabilities to possible candidates for perception.

(4) $$\begin{array}{l} \text{Given }\left[ebko\right]:\\ P\left(ebuko|K_{a}\right)>P\left(ebiko|K_{a}\right)>P\left(ebko|K_{a}\right) \end{array}$$

(5) $$\begin{array}{l} \text{Given }\left[eSpo\right]:\\ P\left(eSipo|K_{a}\right)>P\left(eSupo|K_{a}\right)>P\left(eSpo|K_{a}\right) \end{array}$$

### 1.4. Summary

Although discussed in the previous literature using various terminology, the three main ways that were argued to be the driving factors behind epenthetic vowel selection can be reframed as being motivated by a common goal of rational optimization: Select the output that is most probable given the original input. Epenthesizing the shortest vowel in a given language results in an output that is acoustically the most similar to the original signal, hence is most probable; epenthesizing the vowel with the lowest surprisal in a given context results in an output with total information that is most similar to the original signal, hence is most probable; epenthesizing the vowel that most frequently alternates with zero in a given context results in an output that is representationally equivalent to the original signal, hence is most probable. Note that all three mechanisms are triggered by phonotactic violations, which have extremely high surprisal due to their near-zero probabilities, and are repaired by inserting a segment that consequently removes the locus of high surprisal. Illusory epenthesis, therefore, can also be viewed in information theoretic terms (Shannon, 1948) as smoothing sudden peaks in surprisal. Numerous studies have shown that listeners take longer to process high surprisal (low frequency) words and segments than low surprisal (high frequency) ones (Jescheniak and Levelt, 1994; Vitevitch et al., 1997), suggesting processing difficulties for high surprisal elements. This would suggest that phonotactically illegal sequences that have near-zero probabilities (= near-infinite surprisal) in the listener's language are difficult to process as well. Language users seem to be aware of such processing bottle-necks and have been found to employ various methods to achieve a smoother probability distribution through various phonological manipulations such as syllable duration and prosodic prominence (Aylett and Turk, 2004; Shaw and Kawahara, 2019).

To summarize, there are three main factors involved in illusory vowel epenthesis, all of which are triggered by phonotactic violations in the input and share the goal of selecting the most probable, phonotactically legal alternative as the output. However, the respective contributions of each factor are difficult to tease apart due to a number of assumptions in the previous studies that often have not been tested explicitly. The present study, therefore, investigates the main assumptions behind each of the three epenthesis methods through a series of corpus analyses. The results show that no single method is able to fully account for the observed epenthetic patterns in language users. Section 2 first presents a simulation of how phonotactic restrictions might be learned by a Japanese learner and also describes the Corpus of Spontaneous Japanese, which is used for all simulations and calculations in this paper. Section 3 then discusses in information theoretic terms how the illusory vowel is chosen at a sublexical level according to the phonetic minimality and phonotactic predictability accounts. Section 4 simulates how a Japanese learner might build a lexicon based strictly on surface forms and consequently learn phonological alternations that contribute to illusory vowel epenthesis. Section 5 concludes the study, first by summarizing the overall results and discussing possible avenues for how the different factors involved in illusory vowel epenthesis might be unified into a single system based on convergent proposals from multiple lines of research, ranging from acquisition studies to psycholinguistics and theoretical phonology.

## 2. Phonotactic Learning

Although previous studies generally agree that the process of perceptual epenthesis is the result of repairing phonotactically illegal consonant clusters, Japanese actually allows numerous consonant clusters on the surface. Japanese has a highly productive high vowel devoicing process, where high vowels [i, u] lose their phonation between two voiceless consonants (Fujimoto, 2015). Although devoiced vowels were traditionally analyzed as only losing their phonation while maintaining their oral gestures, recent studies show that there is often no detectable trace of devoiced vowels both acoustically (Ogasawara, 2013; Whang, 2018) and articulatorily (Shaw and Kawahara, 2018). This presents an interesting puzzle whereby Japanese listeners are frequently exposed to and produce consonant clusters, yet repair such sequences with epenthetic vowels during perception. Therefore, rather than assuming that consonant clusters are illegal in Japanese *a priori*, this section first establishes that phonotactic restrictions against heterorganic consonant clusters in Japanese can be learned from the data, using the Corpus of Spontaneous Japanese.

### 2.1. The Corpus of Spontaneous Japanese

All calculations in the present study are based on a subset of the Corpus of Spontaneous Japanese (CSJ; Maekawa and Kikuchi, 2005). The corpus in its entirety consists of ~7.5 million words—660 h of speech—recorded primarily from academic conference talks. The subset used is the “core” portion of the corpus (CSJ-RDB), which contains data from over 200 speakers, comprising ~500,000 words—45 h of recorded speech—that have been meticulously segmented and annotated with the aim to allow linguistic analyses from the phonetic level to the semantic level. The most relevant annotations for the present study are the “prosodic,” “word,” and “phonetic” level annotations. From the prosodic level, the present study primarily uses the intonational phrase for modeling phonotactic learning based on previous findings that infants as young as 6-months of age use prosodic boundary cues to segment clauses and words within speech streams (Jusczyk et al., 1993; Morgan and Saffran, 1995), which suggests that prosodic boundaries can be detected and used for linguistic processing even by the most naïve of listeners. The phonetic level is used for phonotactic learning as well as for calculating predictability in this section. The word and phonetic levels are used together in section 4 to build a lexicon, which is necessary for alternation learning. The word level provides Japanese orthographic representations of all words in the corpus as well as their syntactic categories (e.g., noun, verb, adjective, etc.). The phonetic level provides phonetically detailed transcriptions of the recorded speech, and crucially, indicates the voicing status of vowels.

Two modifications were made to the phonetic transcriptions provided by the CSJ-RDB before using the data as input for calculations. First, “phonetically palatalized” (e.g., /si/ → [s^j^i]) vs. “phonologically palatalized” (e.g., /s^j^a/ → [s^j^a]) consonants, which the CSJ-RDB distinguishes for coronal and dorsal consonants, were collapsed as belonging to the same palatalized consonant. Phonetically palatalized consonants occurred exclusively before /i, iː/, which suggests that the purpose of phonetically palatalized annotations was to reflect coarticulation, where coronal consonants become backed while dorsal consonants become fronted toward a following high front vowel. However, this meant that phonetically palatalized consonants all had near-zero surprisal because only short [i] and long [iː] occurred after these consonants, and the short vowel is over 30 times more frequent than its long counterpart. Furthermore, although the phonetic/phonological distinction might be meaningful underlyingly, it is unclear how phonetically palatalized and phonologically palatalized consonants would differ meaningfully on the surface. Since all of the analyses of the present study assume that phonological learning begins without a lexicon and by extension without knowledge of underlying forms, the difference in palatalization was removed as unlikely to be salient to an uninformed listener.

Second, vowels transcribed as devoiced in the CSJ-RDB were deleted at a probability of 0.10. Recent experimental results show that there is often no detectable acoustic cue (Ogasawara, 2013; Whang, 2018) or articulatory gesture (Shaw and Kawahara, 2018) for vowels that have undergone devoicing, suggesting deletion. However, the CSJ-RDB never transcribes devoiced vowels as deleted. Instead, the CSJ consistently transcribes devoiced vowels as being part of the preceding consonant. For example, the final high vowel in the formal declarative copula *-desu* has a high devoicing rate, and the devoiced copula is segmented as [d], [e], [s$\underset{\circ }{\text{u}}$], where the fricative and the devoiced vowel are segmented together. This shows that the annotators could not reliably separate the devoiced vowel from the preceding consonant but also that the vowel was assumed to be present. It is difficult to conclude with confidence that such unseparated segmentations indicate deletion, however, since there are multiple possible reasons for the annotators' reluctance to mark a segment boundary, such as extreme coarticulation between the segments, lack of obvious vowel spectra despite being audible, lack of vowel cue due to deletion, etc. The story is much the same in previous experimental studies. Despite there being evidence that devoiced vowels do delete, it is difficult to calculate the exact deletion rates due to limitations in the methodology (e.g., reliance on a single acoustic cue to determine deletion; Whang, 2018) or stimuli used (e.g., focusing on a single vowel in limited contexts; Shaw and Kawahara, 2018). The chosen deletion rate of 0.10 is admittedly arbitrary, but it was chosen to introduce some deletion in the data while limiting the number of changes to the original transcriptions that lack clear empirical support. Calculations were also run with deletion probabilities as high as 0.30, but the results were qualitatively similar.

### 2.2. Learning From Unsegmented Speech

The phonotactic learner is based on the Frequency-Driven Constraint Induction mechanism of StaGe (Adriaans and Kager, 2010). StaGe is a lexiconless model built for the purposes of word segmentation in continuous, unsegmented speech. The model is lexiconless based on infant language acquisition studies that showed that infants are sensitive to various aspects of the native language, such as phonetic categories (Werker and Tees, 1984; Werker and Lalonde, 1988; Maye et al., 2002) and phonotactics (Jusczyk et al., 1994; Mattys and Jusczyk, 2001) before the age of 1;0 (years;months) and as early as 0;6. Infants around this age have also been shown to be able to extract words from a continuous stream of speech (Jusczyk and Aslin, 1995; Saffran et al., 1996). In other words, infants already have sophisticated knowledge of their native phonology before acquiring a sufficiently detailed lexicon. The present study, therefore, also assumes that phonotactic learning in Japanese begins before a lexicon is formed and applies the learning mechanism to unsegmented intonational phrases rather than words, as annotated in the CSJ-RDB.

The Frequency-Driven Constraint Induction mechanism of StaGe calculates observed/expected ratios (O/E; Pierrehumbert, 1993; Frisch et al., 2004) of all biphones that occur in the input data and induces constraints by setting thresholds on the O/E ratios. O/E ratios compare how often a biphone actually occurs in the data (Observed) to how often each biphone should have occurred if all segments are assumed to have an equal likelihood of combining to form biphones (Expected) by dividing the probability of a biphone (*xy*) divided by the product of the summed probability of all biphones beginning with (*x*) and the summed probability of all biphones ending with (*y*). The resulting value indicates the magnitude of a given biphone's over-/underrepresentation. For example, O/E ratio of 1 indicates that a given biphone occurred exactly as often as expected, while O/E of 3.0 indicates that a biphone occurred thrice as often as expected.

(6) $$\begin{array}{l} \frac{O\left(xy\right)}{E\left(xy\right)}=\frac{Pr\left(xy\right)}{\sum Pr\left(xY\right)*\sum Pr\left(Xy\right)} \end{array}$$

StaGe induces markedness constraints that flag a biphone as requiring repair for underrepresented biphones. StaGe also induces Contiguity constraints that keep biphones unchanged for overrepresented biphones. The strength of the induced constraints are the target biphones' expected probabilities *E*(*xy*). The thresholds for under- and overrepresentation are arbitrary (perhaps language-specific), but in the original study, Adriaans and Kager (2010) set the O/E thresholds at 0.5 or lower for underrepresentation and 2.0 or higher for overrepresentation. For the present study, the thresholds are set at 0.75 for underrepresentation and 1.25 for overrepresentation so that the model induces constraints more aggressively.

To illustrate the phonotactic learning mechanism of StaGe, suppose that the model receives the following words as input: [k$\underset{\circ }{\text{u}}$to:, kta]. Focusing on the word-initial biphones, the model learns by calculating observed/expected (O/E) ratios that [k$\underset{\circ }{\text{u}}$, kt] are both likely to occur in the language. However, when the model receives [kubi, kumo, kuɡi, kuʤi] as additional input, the O/E of [ku] increases while the O/E for [k$\underset{\circ }{\text{u}}$, kt] decreases. In this way, the O/E ratios of biphones rise and fall based on the data, and when the O/E ratio of a particular biphone sequence falls below 0.75, the model induces a markedness constraint (e.g., *kt: flag *kt* sequence as requiring repair). When the O/E ratio is 1.25 or higher, the model induces a Contiguity constraint (e.g., Contig-*ku*: keep *ku* sequence unchanged). Although it is possible to set constraint induction thresholds based on surprisal instead of O/E ratios, O/E ratios are used as in the original StaGe for the present study. Both surprisal and O/E ratios quantify unexpectedness based on frequency, but there is no obvious reference value for “exactly as expected” for surprisal, whereas this would simply be 1.0 for O/E ratios, making the latter more intuitive to interpret.

### 2.3. Phonotactic Learner Results

Out of 1,280 unique biphones total in the CSJ-RDB, there were 558 consonant-initial biphones. Of them, 127 were overrepresented with O/E ratios >1.25, and 370 were underrepresented with O/E ratios <0.75. The remaining 61 had O/E ratios between the 1.25 and 0.75 thresholds and did not induce constraints. All overrepresented biphones were consonant-vowel biphones, and more importantly all 213 consonant-consonant and 13 consonant-boundary (C#; i.e., word-final consonants) biphones observed in the CSJ-RDB had O/E ratios below 0.75. Shown in Table 1 are five overrepresented consonant-initial biphones with the highest expected values (i.e., the strength of the induced Contiguity constraints that keep the biphone intact) and five underrepresented biphones with the highest expected values (i.e., the strength of the induced markedness constraints that mark the biphone as requiring repair) that illustrate the phonotactic structures that the model learned^1^.

**Table 1:** Five over-/underrepresented biphones in Japanese with highest expected values.

**Overrepresented**			**Underrepresented**		
***xy***	***E(xy)***	***O/E***	***xy***	***E(xy)***	***O/E***
ta	7.61 × 10^−3^	2.21	ti	4.44 × 10^−3^	3.85 × 10^−2^
ka	6.78 × 10^−3^	2.91	tt	3.61 × 10^−3^	2.20 × 10^−3^
to	6.08 × 10^−3^	3.55	kt	3.21 × 10^−3^	4.19 × 10^−2^
ko	5.42 × 10^−3^	2.00	tk	3.21 × 10^−3^	2.91 × 10^−3^
na	4.96 × 10^−3^	3.01	kk	2.86 × 10^−3^	1.16 × 10^−2^

Table 1 shows that overrepresented consonant-initial biphones with the highest expected values in Japanese are all CV. Underrepresented consonant-initial biphones are all consonant clusters with the exception *[ti], which actually reflects another well-known phonotactic restriction in Japanese against high vowels after alveolar obstruents (Ito and Mester, 1995). It should also be noted that coda consonants were distinct from onset consonants in the CSJ-RDB, where [N] represented the placeless nasal coda of Japanese that assimilates in place with the following segment and [Q] represented the first half of a geminate consonant, and thus also placeless. Because the surface forms of [N, Q] are completely predictable based on the following segment, they were left unchanged before the analysis. Therefore, the biphones *[tt, kk] in Table 1 are not geminates but clusters of consonants with independent place features.

The results show that the phonotactic learner learns both a strong preference for CV structure and a strong restriction against CC and C# sequences. However, learning that consonant clusters are prohibited in Japanese is not enough to explain how perceptual repair occurs. StaGe, which the current phonotactic learner is based on, detects phonotactically illicit sequences and inserts a word boundary. However, unlike in the case of the original Dutch data that StaGe was applied to, where many consonant clusters and codas are allowed, simply breaking up a cluster by inserting a word boundary in Japanese would result in C# sequences which are also prohibited. Phonotactic repair requires choosing a vowel to epenthesize when a consonant cluster is detected, which this paper now turns to in the following section.

## 3. Sublexical Factors in Perceptual Epenthesis

In an experimental study, Dupoux et al. (1999) presented Japanese listeners with acoustic stimuli containing the high back rounded vowel [u] of varying durations ranging from 0 to 90 ms occurring between two consonants (e.g., [ebzo] ~ [ebu:zo]). The results showed that Japanese speakers were unable to distinguish vowel-ful tokens from their vowel-less counterparts, erring heavily toward perceiving a vowel between consonant clusters (e.g., [ebzo] → [ebuzo]). The authors proposed that the results are due to the phonotactics of Japanese that disallows heterorganic consonant clusters. This is supported by the phonotactic learner results presented in the previous section, which showed that restrictions against consonant clusters can indeed be learned from the data. The authors further argue that there is a top-down phonotactic effect on perception, where phonotactically illegal sequences are automatically perceived as the *nearest* legal sequence rather than repaired at a higher, abstract phonological level. The nearest legal sequence is one that requires the most phonetically minimal repair, making [u] the best candidate due to its shortness (Beckman, 1982; Han, 1994).

Phonetic minimality captures an important generalization that it is high vowels that tend to be default epenthetic segments cross-linguistically (e.g., [i] in Brazilian Portuguese; [u] in Japanese; and [ɨ] in Korean) and also be targeted for deletion during production. However, reliance on phonetic minimality leads to the prediction that languages can only have one epenthetic segment, unless there are more than one vowel that are equally short. Languages often employ more than one epenthetic vowel for phonotactic repair, as discussed in the introduction. In the case of Japanese, [u] is the most frequent epenthetic vowel, but recent studies by Whang (2019) and Kilpatrick et al. (2020) found that Japanese listeners report hearing [i] instead in contexts where the high front vowel is the most phonotactically predictable.

### 3.1. Calculating Surprisal

Whang (2019) and Kilpatrick et al. (2020) identify the most phonotactically predictable vowel in a given context using surprisal, which is based on the conditional probabilities of vowels after a given consonant [i.e., Pr(*v*∣*C*_1__)]. Surprisal is the negative log_2_ probability, which transforms the probability to bits that indicate the amount of information (effort) necessary to predict a vowel after a given C_1_.

(7) $$\begin{array}{l} -\log_{2}\mathrm{Pr}\left(v|C_{1}\_\right) \end{array}$$

Although the choice of epenthetic vowel by Japanese listeners seems to be affected by phonotactic predictability, both Whang (2019) and Kilpatrick et al. (2020) make a number of assumptions in their calculations that confound surface level phonotactics with underlying representations, which are not subject to phonotactic restrictions. First, though not an assumption in and of itself, the contexts tested are limited depending on the study's focus. Second, as mentioned above, although Whang (2019) calculated the surprisal of vowels after a given consonant, only high vowels were considered after voiceless consonants under the assumption that Japanese listeners must have learned high vowel devoicing already. High vowel devoicing is essentially the reverse of high vowel epenthesis, where the former systematically removes high vowels while the latter recovers them, and thus the two processes most likely affect each other within the Japanese language. However, assuming knowledge of high vowel devoicing *a priori* to explain epenthesis begs the question of how then the devoicing process was learned. Lastly, both Whang (2019) and Kilpatrick et al. (2020) collapse voiced and devoiced vowels as belonging to the same vowel category before calculating surprisal. Indeed devoiced vowels are considered allophones of voiced vowels in Japanese (Fujimoto, 2015) and belong to the same underlying phonological category as their voiced counterparts (e.g., [u, $\underset{\circ }{\text{u}}$] → /u/), but underlying categories are also something that must be learned from alternations in the lexicon. Furthermore, underlying forms are not subject to phonotactic restrictions, which strictly apply to surface structures (Ito, 1986, *et seq*.). Previous infant studies suggest that phonotactic violations are learned at the surface level prior to detailed lexical acquisition (Jusczyk et al., 1994; Mattys and Jusczyk, 2001), and thus necessarily prior also to alternation learning (Tesar and Prince, 2007). In other words, the studies on phonotactic predictability are conflating the effects of phonotactic predictability and phonological alternations, and it is necessary to recalculate phonotactic predictability without collapsing devoiced and voiced vowels in order to tease apart the effects of the two types of phonological knowledge.

### 3.2. Sublexical Surprisal Results

Using the same pre-processed data from the CSJ-RDB as with the phonotactic learner, surprisal was calculated for all vowels after all consonants in the data. The results, shown in Table 2, reveal that the phonotactic predictability account regarding the choice of epenthetic segment in Japanese is only partially upheld once assumptions of higher phonological knowledge is removed. As discussed above, devoiced and voiced vowels were kept distinct, and since Japanese has phonemic vowel length contrasts (e.g., /obasaN/) “aunt” vs. /obaːsaN/ “grandmother”), this resulted in a total of 20 possible vowels: five short voiced [i, e, a, o, u], five short devoiced [$\underset{\circ }{\text{i}}$, $\underset{\circ }{\text{e}}$, $\underset{\circ }{\text{a}}$, $\underset{\circ }{\text{o}}$, $\underset{\circ }{\text{u}}$], five long voiced [iː, eː, aː, oː, uː], and five long devoiced [$\underset{\circ }{\text{i}}$ː, $\underset{\circ }{\text{e}}$ː, $\underset{\circ }{\text{a}}$ː, $\underset{\circ }{\text{o}}$ː, $\underset{\circ }{\text{u}}$ː]. In the interests of space, below are the three vowels with lowest surprisal values after every obstruent consonant observed in the data.

**Table 2:** Vowels with the three lowest surprisal after all obstruents observed in the CSJ-RDB, in order of increasing surprisal.

	**Context**	**Vowel**	**Surprisal**	**Vowel**	**Surprisal**	**Vowel**	**Surprisal**	**Target**
Stops	p	a	1.628	u	2.273	aː	3.275	u
	t	o	1.476	e	1.800	a	1.835	o
	k	a	1.439	o	2.301	u	2.711	u
	b	u	1.502	a	1.800	e	3.127	u
	d	e	0.717	a	2.579	o	2.710	o
	ɡ	a	0.623	o	2.494	e	3.751	u
Affricates	ʦ	u	0.670	$\underset{\circ }{\text{u}}$	1.728	u:	4.832	u
	dz	u	1.441	e	2.166	a	2.589	u
Fricatives	ɸ	u	1.298	$\underset{\circ }{\text{u}}$	1.654	uː	2.541	u
	s	$\underset{\circ }{\text{u}}$	1.918	a	2.600	u	2.627	u
	h	a	1.162	o	1.936	oː	2.395	–
	p^j^	oː	0.277	uː	3.558	a	4.143	i
Palatalized	k^j^	i	0.801	$\underset{\circ }{\text{i}}$	2.351	oː	3.649	i
stops	b^j^	oː	0.517	uː	2.687	a	2.821	i
	ɡ^j^	i	0.664	oː	2.006	a	3.776	i
Palatalized	ʧ	i	1.078	$\underset{\circ }{\text{i}}$	2.810	oː	3.173	i
affricates	ʤ	i	1.023	oː	2.529	uː	2.982	i
Palatalized	ʃ	$\underset{\circ }{\text{i}}$	1.117	i	2.003	o	4.175	i
fricatives	ç	$\underset{\circ }{\text{i}}$	1.214	i	1.844	oː	2.714	i
Non-standard	t^j^	uː	0.561	u	1.710	$\underset{\circ }{\text{u}}$	5.907	–
	d^j^	u	0.485	uː	1.807	–	–	–
	k^w^	a	0.000	–	–	–	–	–
	ɸ^j^	uː	0.00	–	–	–	–	–
	β	a	1.000	i	1.000	–	–	–

The consonants in the “non-standard” rows are atypical, occurring only in loanwords, and are not (yet) regarded as phonemic in Japanese. These non-standard consonants [t^j^, d^j^, k^w^, ɸ^j^, β] each occurred 360, 7, 1, 1, and 2 times, respectively, in the entire CSJ-RDB, and thus are excluded from discussion for the remainder of this paper.

Starting with the stop consonants, Table 2 shows that based on phonotactic predictability the only context in which the “default” [u] would be epenthesized is after [b]. The epenthetic vowel after [p, k, ɡ], [t], and [d] are predicted to be [a, o, e], respectively. In the case of [p, k, ɡ], previous studies have shown repeatedly that it is in fact [u] that is epenthesized after these consonants (Dupoux et al., 1999, 2011; Whang, 2019; Kilpatrick et al., 2020). The results also show that neither [u] nor [i] are predicted to be epenthesized after the coronal stops [t, d]. Instead, [o] is predicted after [t] and [e] after [d]. In fact, high vowels are prohibited in the native and Sino-Japanese lexical strata of Japanese, and it is most often [o] that is epenthesized after coronal stops in loanwords (e.g., /faɪt/ → [ɸaito] “fight”; Ito and Mester, 1995). However, despite the expectation that the illusory vowel should then be [o] in these contexts, this is not borne out in experimental results. Monahan et al. (2009) tested precisely the issue of illusory epenthesis in coronal stop contexts and found that (i) Japanese listeners do not confuse tokens such as [e{t/d}ma] with [e{t/d}uma] but also that (ii) Japanese listeners do not confuse tokens such as [e{t/d}ma] with [e{t/d}oma] either, suggesting that unlike [u, i], the mid-back vowel [o] does not participate in illusory epenthesis. The authors propose that perhaps in coronal stop contexts, Japanese listeners represent the input as [etVma], which is distinct from both [etuma] and [etoma]. Additionally, older loans with coronal stop codas also do not show [o] epenthesis, opting instead for deletion (e.g., /pɑkɛt/ → [pokke ] “pocket”) or [u] epenthesis, which also triggers spirantization (e.g., /waɪt ʃɜɹt/ → [waiʃaʦu] “white shirt”; Smith, 2006). Although loanwords are not the focus of this paper, it seems worth pointing out that the phonotactic calculations and the available experimental evidence suggest that the prevalent use of [o] after [t] in loanwords is not due to illusory epenthesis but possibly due to surface phonotactics of Japanese^2^. This does mean, however, that the phonotactic account fails to predict what Japanese listeners actually perceive in this context as there is no option to posit a featureless vowel. Furthermore, unlike in the case of [to], there is little support for the predicted epenthesis of [e] after [d] in the literature except for the occasional substitution of high vowels with [e] after coronal stops in older loanwords (e.g., /k'akt'uki/ → [kakuteki] “Korean radish kimchi”; /stɪk/ → [sutekki] “(walking) stick”). Whether tokens such as [e{t/d}ema] are perceptually confused with [e{t/d}ma] remains to be tested rigorously.

Setting aside [h]^3^, a surprising result is found with the fricatives. The vowel with the lowest surprisal after [s] is the *devoiced* vowel [$\underset{\circ }{\text{u}}$]. Voiced [u], in fact, is the third most common vowel after [s], leading to the prediction that [$\underset{\circ }{\text{u}}$] would be epenthesized in this context. Aside from [p^j^, b^j^]^4^, Table 2 additionally shows that the phonotactic predictability account would correctly predict the epenthesis of a short high front vowel after palatalized obstruents (Dupoux et al., 1999; Whang, 2019; Kilpatrick et al., 2020). However, as was the case with [s], it is a devoiced vowel that is predicted to be epenthesized after the palatal fricatives [ʃ, ç].

Although phonetic minimality correctly predicts the epenthesis of [u] after non-palatalized consonants [p, k, b, ɡ, ʦ, dz, ɸ, s], it is completely unable to account for the consistent epenthesis of [i] after palatalized consonants. Phonotactic predictability, on the other hand, is able to account for the epenthesis of [i] after palatalized consonants (and perhaps also the non-illusory epenthesis of [o] in loanwords). However, it is a poor predictor for the epenthesis of [u] after non-palatalized consonants once assumptions regarding higher phonological knowledge of underlying forms and high vowel devoicing are removed. In short, both phonetic minimality and phonotactic predictability are unable to fully account for human perceptual epenthetic behavior.

## 4. Learning Alternations From the Lexicon

As shown in Section 3 above, phonetic minimality and phonotactic predictability are both only partially successful in predicting the perceptual epenthesis patterns shown in language users. Here, the present study proposes that the limited success is due to reliance on sublexical, phrase-level phonology. This section shows that a lexicon is necessary to fully account for perceptual epenthesis in Japanese, and more specifically phonological alternations that can only be learned by comparing surface forms that map to the same meaning. Durvasula and Kahng (2015) showed the necessity of phonological alternations in explaining the perceptual epenthesis patterns of Korean listeners, and the parallel between the Korean account in Durvasula and Kahng (2015) and Japanese perceptual epenthesis is not difficult to see. Just as certain vowels regularly alternate with zero in Korean due to productive phonological processes, alternations between high vowels and zero should also be observed in the Japanese lexicon due to the productive process of high vowel devoicing. This section aims to first establish that vowel-zero alternations with a bias toward vowel-fulness can in fact be learned from a lexicon in Japanese, despite there being surface clusters that result from high vowel devoicing/deletion.

### 4.1. Building the Lexicon

A lexicon allows a language learner to keep track of what input forms correspond to what meaning (Apoussidou, 2007) and eventually acquire a paradigm over the lexicon. To learn alternations from a lexicon, one must first build a lexicon, and for a lexicon to be built with sufficient detail, it is necessary to differentiate meaning. To simulate meaning-based learning, the lexicon builder built for the present study relies on a combination of the orthographic representation and syntactic category of each word as provided by the CSJ-RDB. When the lexicon builder encounters a new word, it creates a new lexical entry with the orthographic form, the syntactic category, and phonetic form of the word. Note that the phonetic forms were the same, pre-processed transcriptions from the CSJ-RDB used for the phonotactic analysis, which included deleted vowels. Every time the same combination of orthographic form and syntactic category is encountered, it adds the phonetic form to the entry. If the same phonetic form was encountered before, the lexicon builder simply updates the count. If a new phonetic form is encountered, it starts a separate count for the new phonetic form. A toy example of a resulting lexicon is shown in Table 3.

**Table 3:** Toy lexicon.

**Word**	**Category**	**Gloss**	**Surface**
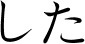	Verb	“did”	[ʃ$\underset{\circ }{\text{i}}$ta] (x7), [ʃta] (x2), [ʃita] (x1)
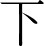	Noun	“down”	[ʃ$\underset{\circ }{\text{i}}$ta] (x4), [ʃita] (x1)
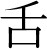	Noun	“tongue”	[ʃ$\underset{\circ }{\text{i}}$ta] (x1), [ʃta] (x1)
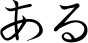	Verb	“exists”	[aru] (x10)
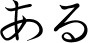	Adjective	“a certain…”	[aru] (x5)

The first three words in Table 3 “did,” “down,” and “tongue” are homophonous, and at least one of each word's phonetic forms overlaps with another word. However, these three words differ in meaning (orthographic forms) and thus are listed as separate entries. This allows the phonetic forms for the words to be counted separately. For example, despite the fact that the words “did” and “down” both occurred with a devoiced [$\underset{\circ }{\text{i}}$] and voiced [i], there are separate counts for the homophonous forms according to lexical entry. Additionally, the last two words “exists” and “a certain…” show why the use of syntactic category was also necessary in building the lexicon. Neither words show any alternation, and thus are completely homophonous; and although they differ in meaning, they have the same orthographic representation. What differentiates them for the lexicon builder in this case is their respective syntactic categories. The lexicon builder learned a total of 14,121 unique words, and of them 3,353 words had more than one phonetic form.

### 4.2. Alternation Learner

With the lexicon established, let us now turn to how phonological alternations might be learned. The lexicon does not yet have underlying phonological representations because it simply mapped one or more phonetic (surface) forms to the same meaning. This was by design as it is the job of the language learner to figure out what single form the alternating phonetic forms must map to. The learner used SequenceMatcher in the difflib package for Python to learn alternations in the lexicon. SequenceMatcher compares two strings (sequences of phones) by setting one as the baseline and identifying substrings in the other to *replace, delete, insert*, or keep *equal* in order to match the baseline. The baseline was always set to the most frequent phonetic form for a given lexical entry. For example, using the entry “did” in Table 3 above, the baseline would be [ʃ$\underset{\circ }{\text{i}}$ta] since it occurred seven times out of 10. SequenceMatcher then would compare [ʃta] and [ʃita] to the baseline and learn the following:

- With [ʃ$\underset{\circ }{\text{i}}$ta] as baseline and [ʃta] as alternate…
  - Keep *equal* the initial segment [ʃ].
  - *Insert* [$\underset{\circ }{\text{i}}$] in the second position.
  - Keep *equal* the third segment [t].
  - Keep *equal* the final segment [a].
- With [ʃ$\underset{\circ }{\text{i}}$ta] as baseline and [ʃita] as alternate…
  - Keep *equal* the initial segment [ʃ].
  - *Replace* the second segment [i] with [$\underset{\circ }{\text{i}}$].
  - Keep *equal* the third segment [t].
  - Keep *equal* the final segment [a].

Each *replace, delete, insert*, or *equal* operation was multiplied by the number of times the alternate form occurred. The baseline was also compared to itself and multiplied by its token frequency. In cases where the alternate form had the same frequency as the baseline, SequenceMatcher was run again with the baseline and alternate forms switched. This was to ensure that the model gives equal weight to lexical alternations with the same probability and does not learn an accidental bias introduced by the sorting method of a particular programming language. Additionally, words that did not alternate were also compared to themselves and multiplied by their respective token frequencies. Multiplying each operation by token frequencies meant that the alternation learner actually learns a bias toward keeping segments unchanged, (i) since only 3,353 words of the 14,121 total showed alternations and (ii) since it is rarely the case that the baseline and alternate forms are completely different. Lastly, since the purpose of this alternation learner was to investigate what alternations can be learned after a given consonant à* la* Durvasula and Kahng (2015), the operations were contextualized with the previous segment. For word-initial segments, the context was a word boundary. For example, the [i] replacement operation above was recoded as [ʃ]:[i] → [$\underset{\circ }{\text{i}}$] (when [i] occurs after [ʃ], replace with [$\underset{\circ }{\text{i}}$]). For every observed *x*:*y*→*z* alternation, surprisal was calculated for the *y*→*z* operation with *x* as the context, quantifying how unexpected it is for the phonological grammar to perform a particular *replace, delete, insert*, or keep *equal* operation after a given consonant.

(8) $$-\log_{2}\mathrm{Pr}(\emptyset \to v|C_{1}\_)$$

Shown in Table 4 are the zero-to-vowel alternations that the model actually learned for every obstruent context. The results of the alternation learner shows that the model correctly learns the necessary alternations between zero and high vowels in almost all relevant contexts. Of equal importance is that because the alternation learner tends to learn a “keep *equal*” bias, the surprisal for vowel-to-zero (deletion) operations are often the highest among all learned operations in a given context. In short, alternation learning strengthens the phonotactic prohibition of consonant clusters in Japanese.

**Table 4:** Nothing-to-vowel alternations with the three lowest surprisal after all obstruents observed in the CSJ-RDB, learned by the alternation learner.

	**Context**	**Vowel**	**Surprisal**	**Vowel**	**Surprisal**	**Vowel**	**Surprisal**	**Target**
Stops	p	u	1.000	$\underset{\circ }{\text{u}}$	1.737	i	3.322	u
	t	o	0.941	e	2.093	a	2.159	o
	k	$\underset{\circ }{\text{u}}$	0.900	u	1.567	a	4.042	u
	b	u	0.807	a	1.807	i	2.807	u
	d	e	0.142	a	4.000	o	6.000	o
	ɡ	u	1.322	a	1.322	–	–	u
Affricates	ʦ	$\underset{\circ }{\text{u}}$	0.812	u	1.216	–	–	u
	dz	u	0.000	–	–	–	–	u
Fricatives	ɸ	$\underset{\circ }{\text{u}}$	0.283	u	2.605	–	–	u
	s	$\underset{\circ }{\text{u}}$	0.110	u	4.705	o	6.095	u
	h	a	0.678	o	1.415	–	–	–
	p^j^	–	–	–	–	–	–	i
Palatalized	k^j^	$\underset{\circ }{\text{i}}$	0.826	i	1.228	a	8.388	i
stops	b^j^	–	–	–	–	–	–	i
	ɡ^j^	–	–	–	–	–	–	i
Palatalized	ʧ	i	0.795	$\underset{\circ }{\text{i}}$	1.455	o	5.087	i
affricates	ʤ	i	0.453	$\underset{\circ }{\text{i}}$	3.700	u	4.700	i
Palatalized	ʃ	$\underset{\circ }{\text{i}}$	0.177	i	3.471	$\underset{\circ }{\text{u}}$	5.910	i
fricatives	ç	$\underset{\circ }{\text{i}}$	0.131	i	3.617	a	7.524	i
Non-standard	t^j^	–	–	–	–	–	–	–
	d^j^	–	–	–	–	–	–	–
	k^w^	–	–	–	–	–	–	–
	ɸ^j^	–	–	–	–	–	–	–
	β	–	–	–	–	–	–	–

It should be pointed out that the alternation learning model predicts that a devoiced high vowel will be epenthesized after most voiceless consonants [k, ʦ, ɸ, s, k^j^, ʃ, ç]. The exceptions are [p, ʧ], after which voiced high vowels are predicted to be epenthesized, and [t, d], after which [o, e] are again predicted to be epenthesized, respectively, as with the phonotactic predictability results. The prediction for devoiced vowel epenthesis after [k, ʦ, ɸ, s, k^j^, ʃ, ç] is supported by Ogasawara and Warner (2009), who found in a lexical judgment task that Japanese listeners have shorter reaction times when presented with devoiced forms of words where devoicing is typically expected compared to when presented with voiced forms. This suggests that Japanese listeners do not restore devoiced vowels as underlyingly voiced for lexical access, relying instead on the more common surface form (Cutler et al., 2009; Ogasawara, 2013). Additionally, the alternation learner predicts that [o, e] will be epenthesized after [t, d], respectively, repeating the shortcomings of phonotactic predictability account in explaining illusory epenthesis in these contexts but providing a possible source for the prevalent use of mid vowels where high vowels are prohibited.

There are two contexts in which the alternation learner is unable to predict the epenthetic vowel—after [ɡ] where [a, u] are the only candidates for epenthesis but have the same surprisal, and after [ɡ^j^] where no alternation with zero was observed in the lexicon. The first problem is attributable to the fact that all devoiced vowels, both high and non-high, were assigned the same rate of deletion. However, it seems reasonable to speculate that devoiced high vowels would be deleted at higher rates than devoiced non-high vowels, since high vowels are the ones that are categorically targeted for devoicing (Fujimoto, 2015), whereas non-high vowel devoicing is a more phonetic process that results from the glottis failing to close sufficiently in time (Martin et al., 2014). Implementing higher deletion rates for devoiced high vowels would increase the overall number of alternations of high vowels with zero relative to non-high vowels in the data; but again, in the absence of accurate deletion rates, it is perhaps hasty to implement different deletion rates according to vowel height simply to increase the model's performance. The second problem can be resolved by implementing a generalization mechanism similar to the feature-based approach of StaGe, which would allow both the phonotactic and alternation models to learn that [i] is most frequent after palatalized consonants in general.

## 5. Discussion and Conclusion

The present study investigated the three main ways in which illusory vowel epenthesis has been argued to occur and showed that no single method is able to fully account for the epenthetic behavior of language users. Section 2 first established that phonotactic restrictions against consonant clusters can be learned from Japanese input, despite the language allowing surface clusters due to a productive high vowel devoicing process. Section 3 then discussed the roles of phonetic minimality and phonotactic predictability. Crucially, the section revealed that once the misassumption of access to underlying forms is corrected for, phonotactic predictability is only successful in predicting the epenthesis of [i] in palatal contexts but not the epenthesis of [u] in other contexts. The results also showed that the phonotactic predictability account predicts the epenthesis of mid vowels [o, e] after [t, d], which finds support in the loanword literature but not in the experimental literature. Lastly, Section 4 showed that phonological alternations are the most successful at predicting the identity of illusory vowels in given contexts even with a small number of deletion introduced to the data, but also that it too is unable to account for all contexts. Specifically, it predicts the epenthesis of devoiced high vowels after most voiceless consonants, which although somewhat surprising at first glance is supported by lexical access studies. Additionally, as with the phonotactic predictability account, the phonological alternations learned by the model predict the epenthesis of [o, e] after [t, d]. Lastly, it is unable to narrow down the choice of epenthetic vowel in certain contexts.

The main proposal of the present study was that all three methods for illusory epenthesis can be reframed as having the same rational goal of choosing the optimal epenthetic vowel that results in the smallest amount of change to the original speech signal, motivated by the need to smooth extreme surprisal peaks in the signal that make processing difficult (Jescheniak and Levelt, 1994; Vitevitch et al., 1997; Aylett and Turk, 2004). A phonotactic violation is detected when there is a spike in surprisal caused by a sequence of sounds that are rarely or never adjacent to each other in the listener's native language. In the case of Japanese, the language has a strong CV preference, and thus listeners epenthesize a vowel between illicit consonant-consonant sequences. For example, according to the phonotactic analysis discussed in section 3, when [k] is followed by [t], the surprisal is 8.635, but by epenthesizing [u] between the two consonants, the result is substantial smoothing of surprisal, where the surprisal after [k] is now lowered to 2.711 and the subsequent transition from [u] to [t] is 3.924. Even when the surprisal is summed, the transition from [k] to [t] is now lower than when there was no intervening vowel. A phonetically minimal vowel is one that least alters the acoustic characteristics of the original input, and thus is an optimal repair. Similarly, a vowel that has the highest phonotactic predictability in a given context is one that has the lowest information density, altering the least the total information content of the original input, and thus is an optimal repair. Lastly, epenthesis based on knowledge of phonological alternations moves the search for the optimal vowel for repair from the sublexical domain to the lexical domain. However, the rational motivation remains the same. A vowel that phonologically alternates with zero in a given context is equivalent to zero in that context, and thus epenthesizing the vowel that most frequently alternates with zero least alters the phonological representation of the original input.

The problem as the results in the current paper showed is that the “optimal” vowel can differ depending on the level of analysis. For example, after [k^j^], the optimal vowels are [u, i, $\underset{\circ }{\text{i}}$] according the phonetic minimality, phonotactic predictability, and phonological alternation accounts, respectively. The question, then, is what level takes precedence? There are multiple converging lines of research that suggest that lexically driven processes take precedence over sublexical processes. First, previous phonological literature that have long noted the importance of the lexicon in phonological processing within traditional generative approaches best exemplified by Lexical Phonology (Kiparsky, 1982; Mohanan, 1982, *et seq*.). Although the theoretical details differ slightly between Kiparsky (1982) and Mohanan (1982) and their respective related works, they share the intuition that there are lexical and postlexical levels of phonological processing, where phonological rules (and/or constraints) operate first on underlying, morphological units at the lexical level, the results of which are processed at the postlexical level as combined phrase-sized units. Second, more recent, functional approaches as exemplified by Message-Oriented Phonology (Hall et al., 2016) go a step further and argue that since the main purpose of language is communicating *meaning*, phonological grammars are shaped largely by lexical concerns, where processes that more directly aid lexical access take precedence. Although the motivations differ, generative and functional approaches agree that the lexicon plays a primary role in phonology. Phonological alternations rely on lexical knowledge, and thus Durvasula and Kahng (2015)'s proposal that epenthesis based on phonological alternations must take precedence, where phonetic/phonotactic factors only come into play when there are no strong expectations that arise from knowledge of phonological alternations is also in line with the theoretical literature. In other words, illusory epenthesis is a serial process, starting from phonological processes that require lexical knowledge followed by sublexical processes that rely on surface-level phonotactics and/or phonetic cues.

Another converging line of work on the primacy of the lexicon is exemplified by Mattys et al. (2005), who investigated the interaction of lexical, phonotactic, coarticulatory, and prosodic cues in speech segmentation, all of which have been shown in previous studies to have a significant effect. The results revealed a hierarchical relationship among the different cues, where listeners use sublexical cues only when noise or lack of relevant contexts make reliable use of lexical information difficult. The results further showed that at the sublexical level, segmental cues (phonotactics, coarticulation, etc.) take precedence over prosodic cues. Of particular relevance for the current discussion is that at the sublexical level, the relative “weights” of different segmental cues are proposed to be language-specific, and thus have no set hierarchy. Mattys et al. (2005) do not provide details on how the language specific weights might be calculated, but again there is a diverse body of works from multiple traditions that bear on this issue.

First, the integration of different segmental cues are rather straightforward under a rational framework. Turning back to illusory epenthesis, the optimization process can be formalized as in (10), where *A* denotes the acoustic characteristics of the input and *K*_*p*_ denotes the phonotactic knowledge of the listener.

(10) $$\begin{array}{l} \text{Given }\left[ebko\right]:\\ P\left(ebuko|A,K_{p}\right)>P\left(ebiko|A,K_{p}\right)>P\left(ebko|A,K_{p}\right) \end{array}$$

Indeed, when framed in this way, the question of an integrated sublexical evaluation becomes similar to that of Sonderegger and Yu (2010), who investigated how an optimal listener compensates for vowel-to-vowel coarticulatory effects^5^. The optimal listener does not rely on phonetic minimality (*A*) or phonotactic predictability (*K*_*p*_) alone, which often give conflicting predictions. Rather the listener considers them together to arrive at an output that is optimal, interpreting acoustic-phonetic cues based on prior knowledge of how they vary in certain phonotactic contexts. It should be noted that because the rational account views the choice of output candidates as a consequence of the listener's optimization process, which in turn is based on the listener's prior linguistic experience, the account also predicts different probabilities depending on the listener. With listeners of sufficiently divergent linguistic experiences, the optimal outputs would differ, and thus the integration of multiple cues under a rational framework is also applicable to crosslinguistic perception.

Second Hume and Mailhot (2013) also propose a similar integration of contextual and phonetic information using an information theoretic framework, but additionally point out that it is non-trivial to precisely quantify the informativity of various phonetic cues. In the interests of space, the current paper simply suggests that an information theoretic framework might also be useful in quantifying the relative informativity of a given segment's acoustic/phonetic cues. For example, let us assume the following vowel system: [i, y, u], which can be distinguished along the height ([±high]; F1), backness ([±back]; F2), and roundedness ([±round]; F3) dimensions. Since all three vowels are high (low F1), height cues have very low surprisal and thus are not informative. This leads to the prediction that listeners of this language would be more sensitive to the backness and roundedness cues than to height cues.

Lastly, a more sophisticated view of phonetic minimality that looks inside segments in more detail to precisely quantify and model the informativity of transitional cues seems necessary. The models in this paper and the previous literature on which the models are based on assume that the basic unit of phonological processes is the segment, but various lines of theoretical research such as Aperture Theory (Steriade, 1993), Articulatory Phonology (Browman and Goldstein, 1992; Gafos, 2002, *inter alia*), and more recently Q Theory (Shih and Inkelas, 2014) all have shown that representing segments in more detail results in a substantial increase in a framework's capacity to capture gradient and autosegmental phonological phenomena. The advantages of a more detailed segmental representation is not just theoretical, although it does complement formal approaches to perceptibility effects on phonology (e.g., P-map, Steriade, 2001; Uffmann, 2006). It also affords a more precise way to quantify and model which transitional cues an optimal listener relies on to select an acoustically “minimal” epenthetic segment and also how low-level phonetic information interacts with phonotactic information^6^.

Assuming an optimal perceptual structure, where lexical processes apply first, followed by a language-specific combination of sublexical processes only when the lexical processes fail to choose an optimal output, we now turn back to the issue of [ɡ] and [ɡ^j^]. What is puzzling about the [ɡ] and [ɡ^j^] cases is that the two contexts require different sublexical mechanisms to predict the correct epenthetic vowel. After [ɡ], phonological alternations regard [a, u] as equally likely options for epenthesis. If the vowel is chosen based on phonotactic predictability, the wrong vowel [a] would be chosen, since it is the most phonotactically probable vowel in the given context. So then the target vowel [u] must be chosen based on phonetic minimality in this case. In the case of [ɡ^j^], the situation is reversed. There are no zero-vowel phonological alternations after [ɡ^j^], so all vowels are possible candidates for epenthesis. If a vowel is chosen based on phonetic minimality as with [ɡ], however, the chosen vowel would be incorrect as Japanese listeners perceive [i] in this context (Whang, 2019). So then after [ɡ^j^], the epenthetic vowel must be chosen based on phonotactic predictability. In an integrated system as described above, the decision may be made as follows by an optimal listener. First, the burst noise of *a*-coarticulated [ɡ] and *u*-coarticulated [ɡ] are not only acoustically different, there is also evidence suggesting that Japanese listeners are sensitive to such coarticulatory differences (Whang, 2019). In other words, representing and quantifying the coarticulatory information can help predict the perceived similarity between the [ɡ] burst in a [ɡ]-C sequence and in a [ɡ]-[u] sequence relative to the burst in a [ɡ]-[a] sequence that makes [u] the phonetically minimal epenthetic vowel. Additionally, if the [ɡ] burst in a [ɡ]-C sequence is judged to be similar to an *u*-coarticulated [ɡ], the most phonotactically predictable vowel in this context would naturally be [u], resolving the apparent conflict between the phonetic minimality and phonotactic predictability accounts. The same process applies to [ɡ^j^]. The epenthetic vowel that would result in a C-V transition that is acoustically the most similar to the fronted velar burst of [ɡ^j^] is [i], again corroborating the predictions based on phonotactic predictability. It is perhaps premature to speculate further on the predictions of such an implemented model based on just two contexts. The present study, therefore, simply presents it as an example of how a rational approach can be used to bring together insights from various lines of research to integrate the seemingly contradictory predictions from different levels of linguistic processing, leaving more rigorous investigations for future studies.

## Data Availability Statement

The datasets presented in this study can be found in online repositories. The names of the repository/repositories and accession number(s) can be found at: https://github.com/jdwhang/RAILS-data.

## Author Contributions

The author confirms being the sole contributor of this work and has approved it for publication.

## Conflict of Interest

The author declares that the research was conducted in the absence of any commercial or financial relationships that could be construed as a potential conflict of interest.

## Publisher's Note

All claims expressed in this article are solely those of the authors and do not necessarily represent those of their affiliated organizations, or those of the publisher, the editors and the reviewers. Any product that may be evaluated in this article, or claim that may be made by its manufacturer, is not guaranteed or endorsed by the publisher.

## Footnotes

^1^Full results of all analyses in the present paper can be found in the author's repository, the link to which can be found in the data availability statement.

^2^The full surprisal results show that the [t] context occurred 83,399 times in the CSJ-RDB, of which more than a third (29,983) was the [to] sequence, perhaps due to the frequent use of the homophonous conjunctive and quotative particles /-to/. In other words, the use of [o] as the epenthetic vowel in loanwords in Japanese is grounded in the statistical tendencies of the native phonology, which is in line with other previous research on loan phonology that have argued that seemingly “novel” loanword patterns are actually instantiations of previously “covert” statistical generalizations in the native grammar (Zuraw, 2000; Kubozono, 2006; Rose and Demuth, 2006).

^3^To this author's knowledge, [h] has never been previously tested in the perceptual epenthesis literature because the consonant is susceptible to extreme coarticulation with surrounding segments due to its lack of oral gestures. It is often the allophones of the phoneme /h/, namely [ɸ, ç], which occur before [u, i], respectively, that are included in studies.

^4^For the palatalized consonants, recall that unlike coronal and dorsal consonants, there were no labial consonants that were transcribed as “phonetically palatalized” in the CSJ-RDB. This meant that after [p^j^, b^j^], there were zero instances of high front vowels [i, $\underset{\circ }{\text{i}}$, iː, $\underset{\circ }{\text{i}}$ː].

^5^The author is grateful to the editor for suggesting this connection.

^6^Quantifying (combinations of) cues/features that decide “phonetically minimal” vowels may also provide additional insight into why it is [o] that became the default epenthetic segment after both [t, d] in loanwords despite [e] being the most frequent after [d]. It seems likely that the transition from [d] to [o] is acoustically more consistent with a [d] burst than the transition from [d] to [e]. In the same vain, the transition from [t, d] to [u] should be acoustically even more similar to the stop bursts in consonantal contexts, and it would be interesting to see if [u] eventually replaces [o] as the default epenthetic vowel in loanwords for these contexts as the restriction against [tu, du] continues to weaken in Japanese. More recent loans provide some evidence for this regularization of [u] epenthesis (e.g., [twaɪs] → [tuwaisu] “twice”; [tɹu] → [tuɾu:] “true”; [dwɛliŋ] → [dueɾiŋɡu] “dwelling”), but what this means for illusory epenthesis in these contexts remains to be seen.
